# Statistical Explorations and Univariate Timeseries Analysis on COVID-19 Datasets to Understand the Trend of Disease Spreading and Death

**DOI:** 10.3390/s20113089

**Published:** 2020-05-29

**Authors:** Ayan Chatterjee, Martin W. Gerdes, Santiago G. Martinez

**Affiliations:** 1 Department of Information and Communication Technology, Centre for e-Health, University of Agder, 4604 Kristiansand, Norway; martin.gerdes@uia.no; 2 Department of Health and Nursing Science, Centre for e-Health, University of Agder, 4604 Kristiansand, Norway; santiago.martinez@uia.no

**Keywords:** COVID-19, public health, ICD, community disease, transmission rate, RNN, LSTM, spread factor, population, correlation, regression, python, hypothesis test, keras, measurable sensor data, artificial intelligence, deep learning, machine learning, statistics, algorithm

## Abstract

“Severe Acute Respiratory Syndrome Coronavirus 2 (SARS-CoV-2)”, the novel coronavirus, is responsible for the ongoing worldwide pandemic. “World Health Organization (WHO)” assigned an “International Classification of Diseases (ICD)” code—“COVID-19”-as the name of the new disease. Coronaviruses are generally transferred by people and many diverse species of animals, including birds and mammals such as cattle, camels, cats, and bats. Infrequently, the coronavirus can be transferred from animals to humans, and then propagate among people, such as with “Middle East Respiratory Syndrome (MERS-CoV)”, “Severe Acute Respiratory Syndrome (SARS-CoV)”, and now with this new virus, namely “SARS-CoV-2”, or human coronavirus. Its rapid spreading has sent billions of people into lockdown as health services struggle to cope up. The COVID-19 outbreak comes along with an exponential growth of new infections, as well as a growing death count. A major goal to limit the further exponential spreading is to slow down the transmission rate, which is denoted by a “spread factor (f)”, and we proposed an algorithm in this study for analyzing the same. This paper addresses the potential of data science to assess the risk factors correlated with COVID-19, after analyzing existing datasets available in “ourworldindata.org (Oxford University database)”, and newly simulated datasets, following the analysis of different univariate “Long Short Term Memory (LSTM)” models for forecasting new cases and resulting deaths. The result shows that vanilla, stacked, and bidirectional LSTM models outperformed multilayer LSTM models. Besides, we discuss the findings related to the statistical analysis on simulated datasets. For correlation analysis, we included features, such as external temperature, rainfall, sunshine, population, infected cases, death, country, population, area, and population density of the past three months—January, February, and March in 2020. For univariate timeseries forecasting using LSTM, we used datasets from 1 January 2020, to 22 April 2020.

## 1. Introduction

In December 2019, Chinese authorities released the first official information to the world about the spreading of the human coronavirus in their country as a community disease [1,2,3]. Till 15th May 2020, more than 4.6 million people are infected with more than 0.38 million death cases reported worldwide by WHO [4,5]. 

In frequent cases the COVID-19 disease develops serious symptoms, to older adults and those having medical pre-conditions, such as cardiovascular diseases (CVDs), diabetes, chronic respiratory diseases (COPDs), cerebrovascular disease, and cancer [6]. Most people infected with SARS-CoV-2 are experiencing no or just mild to moderate respiratory symptoms and recover without special treatment. The COVID-19 disease causes primary symptoms such as fever, tiredness, dry cough, shortness of breath, together with other secondary symptoms such as a runny nose, sore throat, aches and pains, diarrhea, and nausea in its victims after two to fourteen days of contact. In a minority of patients, it causes severe pneumonia that can lead to death with comorbidity. SARS-CoV-2 has a high resemblance to the virus in the body of bats (96.2% identical) termed as “BatCoV RaTG13” and pangolin [1,2,6,7]. But its origin is still a mystery. Once exposed to the environment through a droplet from an infected person, it can last from a few minutes to several hours, up to a few days, depending on the type of surfaces. If a person touches their nose or eyes or pulls it out of their breath, there is a possibility of getting infected. Studies show that it can stay in the air for up to three hours [8], on a copper ring up to four hours, a full day on cardboard, and on plastic and stainless steel it can last up to 2–3 days [9]. In the high phase of the epidemic outbreak, each infected person can infect in average around 3–5 other persons [6].

The genome of SARS-CoV-2 is ribonucleic acid (RNA) [6,10]. Because of such genomic pattern, broad diversity, and recurrent recombination of the coronavirus species, it changes its nature frequently with a mutation to cope up with a new environment for survival [6,10]. A preliminary study has estimated the reproductive number or transmission rate (R0) of COVID-19 to be between 1.3 and 3.5, compared to an R0 of seasonal influenza (flu) and SARS of 1.3 and 2.0, respectively. If R0 > 1, then there is a chance of an epidemic development, else it disappears slowly [4,11].

**Figure 1:**
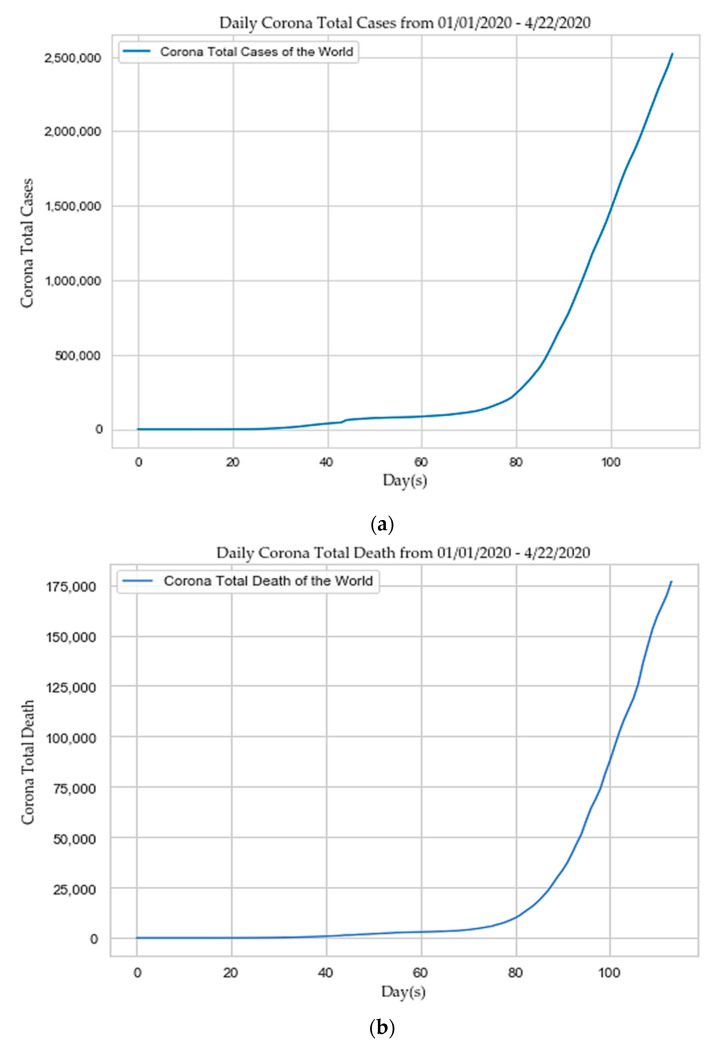
(**a**) Daily corona total new cases; (**b**) and daily corona total death.

Each year an estimated 290,000 to 650,000 people (corresponding to 795 to 1781 per day) die globally due to impacts from flu viruses. SARS was another coronavirus that began from Beijing, China, and spread to 29 countries in between November 2002 to July 2003, afflicting 8096 people, and resulting in 774 fatalities, with a fatality rate of 9.6% [4,6,11]. On 30th January 2020, the case number of the novel coronavirus (SARS-CoV-2) outpaced SARS. Another category of coronavirus, MERS, decimated 858 people out of the 2494 infected, with a fatality rate of 35% in 2012 [6]. COVID-19 outnumbered the fatality rates caused by the other coronavirus categories, such as, SARS [12], and MERS [13], and forced WHO to broadcast a global emergency on 30th January 2020. As proclaimed by WHO on 27th January 2020, the median age of COVID-19 cases diagnosed outside of China was 45 years, ranging from 2 to 74 years, with 71% male incidents. According to China’s “National Health Commission (NHC)”, 80% died victims were over the age of sixty years old, and 75% of them had pre-existing health conditions, such as CVDs and diabetes. According to the “US Center for Disease Control and Prevention”, at least 20% of affected people become so sick that they have to be hospitalized, even though they are between the ages of 20–44, and two-thirds of them are admitted to the “Intensive Care Unit (ICU)”. The rapid growth of COVID-19 across the world from 1 January 2020, to 22 April 2020 is depicted in Figure 1 [4,6,11,14].

COVID-19 is a new ICD code and appeared with multiple significant research questions, research directions, and that is the reason, the research outcome related to COVID-19 is limited in numbers Multiple reputed publishing agencies such as, “Springer”, “Nature”, “Wiley”, “Taylor & Francis Group” have made all the COVID-19 related articles open access and freely available [15,16]. Different studies have been conducted by different research groups on COVID-19 to analyze its nature, effect, spreading, probable consequences with statistical data analysis and AI based approaches. We classified COVID-19 related studies based on two popular AI inspired approaches, such as machine learning (ML) and deep learning (DL) as follows–(a.) machine learning-based approaches-Dong et al. [17] developed an interactive publicly available web-based dashboard to track the outbreak by scientists, researchers, public health authorities, and general people. It was hosted by the “Center for Systems Science and Engineering (CSSE)” at Johns Hopkins University, Baltimore, MD, USA, to visualize and follow reported cases of COVID-19 in real-time. Yang et al. [18] developed a dynamic SEIR model with machine learning (ML) to predict the COVID-19 epidemic peaks and sizes with 2003 data for training after 23 January in China. The research team guessed when the epidemic would be highest in Hubei, China, and when it would start declining gradually, considering quarantine as a factor. Rao et al. [19] did their research on a machine learning (ML) based framework to identify COVID-19 related cases quickly using a phone-based survey. The framework can help to classify cases between no-risk, minimal-risk, moderate-risk, and high-risk, so that high-risk cases can be quarantined earlier, therefore diminishing the chance of spread. Men et al. [20] researched the incubation period of COVID-19 with a machine learning (ML) approach, and their result showed that the incubation distribution of COVID-19 did not follow general incubation distributions such as Lognormal, Weibull, and Gamma distributions. They estimated that the mean and median of COVID-19 incubation were 5.84 and 5.0 days respectively, via bootstrap and proposed “Monte Carlo” simulations. They also noticed that the incubation period of the groups with age >= 40 years and age < 40 years exhibited a statistically significant variation. The initial group had more extended incubation period and more significant variance than the later. The study further indicated that separate quarantine time should be employed to the groups for their distinct incubation periods. Pandey et al. [21] did their research on proactive management with machine learning methods to raise the “WASH” awareness for maintaining personal hygiene. They utilized the co-creation technique to develop the user interface solution using mHealth technologies (WashKaro app) in the local Indian language “Hindi”. They utilized a total of 13 combinations of pre-processing approaches and evaluated word-embeddings, similarity metrics by 8 human participants via calculation of agreement statistics. The archived the best performance with Cohen’s Kappa of 0.54, and the solution was deployed as “On Air”, WashKaro app’s AI-powered back end. Li et al. [22] evaluated the risk of a pandemic for all cities and regions in China using popular machine learning classifier ‘Random Forest (RF)’ with identified factors such as accumulative and increased numbers of confirmed cases, total population, population density, and gross domestic product (GDP). The experiment found a risk of unnecessary economic loss due to COVID-19. Yan et al. [23] and Jia et al. [24] Worked on the predictive model to predict the criticality of COVID-19. The first research group developed a machine learning based (XGBoost) prognostic model with clinical data in Wuhan from 10 January to 18 February 2020, based on 3 clinical features. The model can predict the health risk and quickly access the risk of death. The former research group used the “Logistic model”, “Gompertz model” and “Bertalanffy model” to predict the cumulative number of confirmed cases and the development trend of the COVID-19 epidemic. The “Logistic model” outperformed other models in fitting all the data in Wuhan, while the “Gompertz model” performed better in fitting the data in non-Hubei areas. Randhawa et al. [25,26] conducted two ML-based genomic studies to analyze the genomic signatures to provide evidence of associations between Wuhan 2019-nCoV and bat coronaviruses and to classify novel pathogens of COVID-19 rapidly. (b.) deep learning-based approaches-Gozes et al. [27] developed artificial intelligence-based automated 2D and 3D deep learning-based CT image analysis tools to detect, quantify, track, and monitor corona infected patients from those who have not infected. Zhang et al. [28] proposed a deep learning-based drug screening model “DFCNN” for novel coronavirus 2019-ncov with virus RNA sequence database “GISAID” of Coronavirus and demonstrate that they can differentiate coronavirus patients from those who do not have the disease. Xu et al. [29] conducted a study to establish an early screening model to distinguish COVID-19 pneumonia from Influenza-A viral pneumonia and healthy cases with pulmonary CT images using deep learning techniques with 86.7% accuracy. Shan et al. [30] and Li et al. [31] conducted their research on CT images with deep learning techniques to quantify lung infections in a COVID-19 patient and to distinguish COVID-19 patients from community-acquired pneumonia patients, respectively. Narin et al. [32] and Wang et al. [33] did their research on Deep Convolutional Neural Network Design to identify the COVID-19 cases from the chest X-ray images. Ghosal et al. [34] investigated on drop-weights based “Bayesian Convolutional Neural Networks (BCNN)” to guesstimate uncertainty in deep learning-based solution to expand the diagnostic performance of the human-machine team using publicly available COVID-19 chest X-ray dataset and exposed that the uncertainty in prediction is highly correlated with the accuracy of prediction. Santosh et al. [35] and Hu et al. [36] did their research on the human coronavirus outbreak forecast model with AI approaches. The former research group utilized ML algorithms to analyze data and followed by decision making to forecast the nature of COVID-19 spread across the globe using active learning-based cross-population train/test models that used multimodal data. The following research group used deep learning LSTM model (modified stacked auto-encoder) to forecast and estimate the size, lengths, and ending time of COVID-19 across China based on the data collected from January 11 to 27 February 2020, by WHO. Maghdid et al. [37] designed an AI-enabled framework to diagnose COVID-19 using smartphone embedded sensors. The developed low-cost solution takes input from the camera sensor (CT scan images of lungs, human tracking video observation), inertial sensor (30-second-sit to stand), microphone sensor (cough voice prediction), temperature fingerprint sensor (fingerprint on the screen) to predict COVID-19 disease, based on the deep learning (RNN and CNN) techniques. 

The AI inspired approaches are a powerful tool for helping public health planning and policymaking. Our research aims to perform statistical analysis on available COVID-19 related datasets available in “ourworldindata.org” [5] and newly created dataset to find a set of probable risk factors associated with the spreading of COVID-19 and we have identified it as a research gap. Once correlation analysis was accomplished, we explored univariate LSTM models for timeseries forecasting on total cases and deaths. LSTM is an artificial “recurrent neural network (RNN)” architecture and used in the field of deep learning. Therefore, in this research we followed deep learning-based approach. In addition, we proposed an algorithm to prove our assumed hypothesis that that social isolation or social distancing might restrict the spreading of the COVID-19.

The global scientific community is looking for three possible solutions, such as virus enzyme inhibitors [38,39], plasma therapy [40], and vaccination to give a counter fight against COVID-19. According to the WHO director general, the safest and fastest method of corona treatment is patient identification, separation, examination, and treatment. WHO has specified a standard on its official website where guidelines are specified formally to slow down and prevent its further transmission. “Worldometers.org” [11], “ourworldindata.org” [5], and WHO [4] are updating situation reports, data tables, and a COVID-19 dashboard on regular basis. We assumed that all the available data provided by all countries on total case numbers, total deaths, total recoveries, daily cases, daily deaths, and daily recoveries are correct, and based on that assumption we carried out our further analysis of the data.

The main contributions of this paper are as follows: (a) Risks associated with the human coronavirus spreading? (b) Identification of a set of probable correlated factors associated with the expansion of COVID-19 following statistical approaches on the fabricated datasets? (c) Analysis of the impact of social isolation with a spread factor (*“f”*) to restrict the spread of the human coronavirus? (d) Analysis of different univariate LSTM models for forecasting of total cases and total deaths caused by COVID-19.

The remainder of the paper is structured as follows: In Section 2, risks associated with the human coronavirus spreading is discussed with data. Section 3 describes the methodology utilized for the data processing. In Section 4, we discuss our findings. The paper is concluded in Section 5. Clinical trials, chemical compounds, genetic analysis, political arguments, and economic analysis related to COVID-19, are beyond the scope of this paper.

## 2. Risks Associated with the Spreading of COVID-19

COVID-19 has created significant health and economic slowdown in many countries since January 2020 due to global and local lockdown to encourage social distance. It has infected more than 4.6 million people so far, with more than 0.38 million death and more than 1.7 million recoveries reported until 15th May 2020 [4,5]. It attacked not only developed nations but also developing ones, regardless of the socioeconomic condition, age, and gender discrimination. COVID-19 is highly contagious and transmissible from human to human, with an incubation period of up to 24 days [6].

WHO officials initially considered SARS-CoV-2 as non-airborne, but a recent study has discovered that it can survive in air staying suspended as aerosol depending on factors such as heat and humidity [41]. Therefore, the infection mediums can be classified as contact (direct or indirect), droplet spray in short-range transmission, and aerosol in long-range transmission (airborne transmission) [41]. According to the “Center for Disease Control and Prevention (CDC)”, a social distance of about 1.8 m is necessary to avoid large droplets of virus-laden mucus [42], but some experts suggest that 1.8 m distance is not enough [43] due to possible air current (Table 1). Pollution caused by nitrogen dioxide (NO_2_) can be one of the most critical contributors to increase the fatality rate, caused by COVID-19 [44]. Recent studies found the existence of SARS-CoV-2 in sewage water [45] and non-potable water [46]. 

Scientists are exploring how humidity, temperature, and ultraviolet lighting alters the virus as well as how long it can survive on different surfaces. Some studies have revealed that relative humidity affects all infectious virus droplets, independent of their source and location [41], and gravity and airflow cause the most virus droplets to float to the ground. The temperature, along with humidity, affects the properties of viral surface proteins and lipid membrane [41]. According to the same study, humidity between 50% to 80% is the best for low stability in SARS-CoV-2 [41]. According to the studies [9,53,54], The SARS-CoV-2 can exist on different objects and surfaces as follows: (a) half of the samples from the soles of the ICU medical staff shoes tested positive, (b) surface contamination (computer mouse, trash cans, sickbed handrails, doorknobs), (c) equipment (exercise equipment, medical equipment including spirometer, pulse oximeter, and nasal cannula, personal computers, iPads, and reading glasses), and (d) surfaces (cellular phones, remote controls, toilets, room floors, bedside tables, and bed rails, and window ledges). 

**Figure 2:**
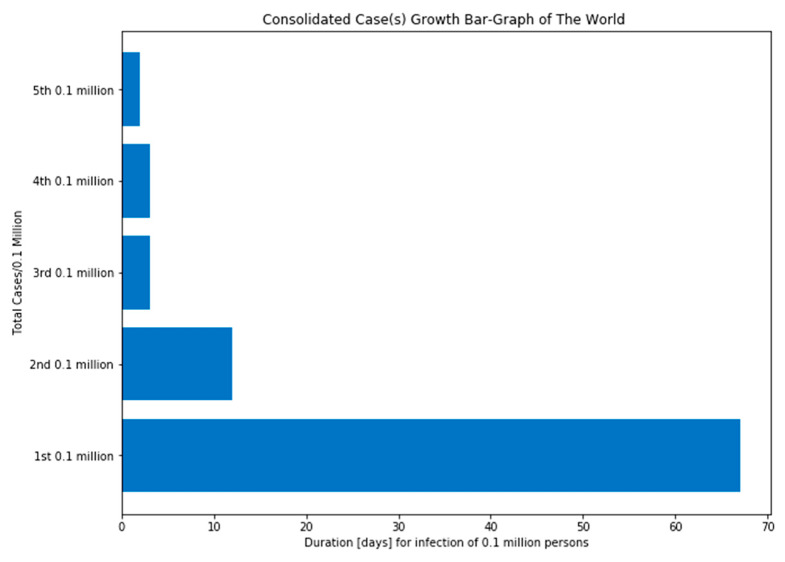
Consolidated case(s) growth of the “world”.

According to the epidemiologists, the fatality rate of COVID-19 can change as SARS-CoV-2 can mutate. WHO claimed that social distancing is the only way to slow down COVID-19 transmission, and that is the reason, many countries are locked down, and people are asked to stay at home. The concept of social distancing is not to eradicate the COVID-19, but to slow down its transmission, hence declining the pressure on the health care systems and economy and, in this manner, reduce the fatality rate. It might infect around 90% of the global population if no mitigation measures are taken soon, as estimated by a leading statistical modeling group at “Imperial College London (ICL)” [55]. COVID-19 took 67 days for its initial 0.1 million cases, then it took just 3 days to reach from 0.4 million to 0.5 million cases as depicted in Figure 2. The ICL team analyzed that, if proactive measures, such as social distancing, rigorous testing and isolation of diseased people are taken with proper planning when fatality rate of each infected country is 0.2/100,000 victims/week, then the outcome might reduce wide-reaching deaths to 1.9 million. Studies found that Italy hit the 0.2 threshold on 2nd–3rd March, the United Kingdom on March 17, and the United States on March 22 [4,5]. 

**Figure 3:**
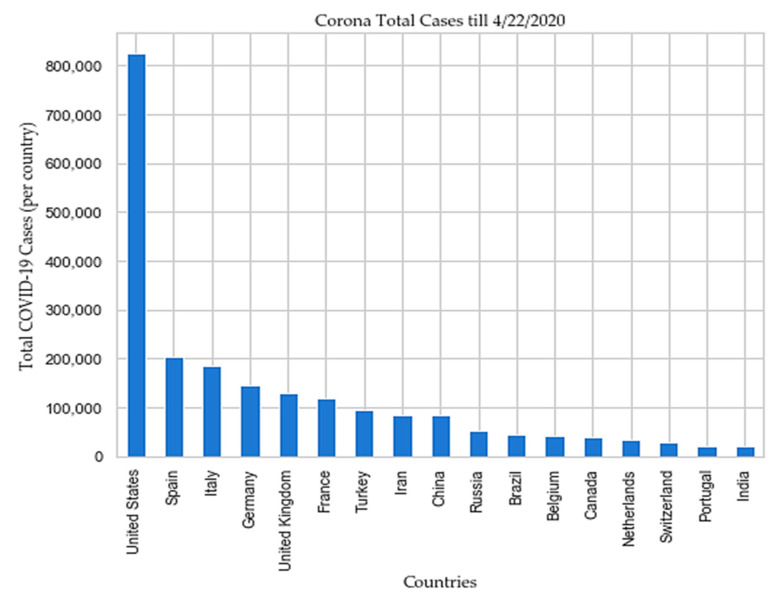
Top 17 countries according to the total cases reported till 22 April 2020.

**Figure 4:**
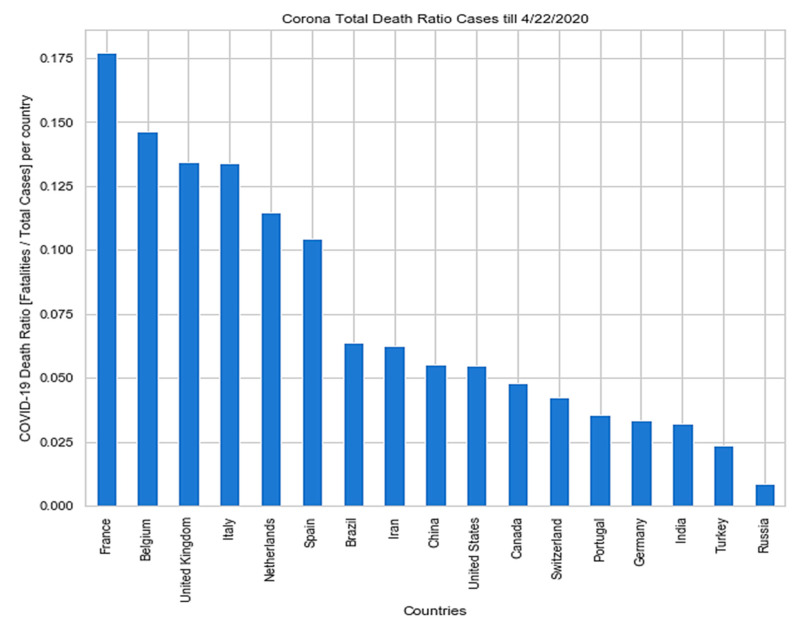
The death ratio of top 17 countries according to the total cases reported till 22 April 2020.

USA, Spain, Italy, France, Germany, UK, Turkey, Iran, China, Russia, Brazil, Belgium, Canada, Netherlands, Switzerland, Portugal, and India are top 17 countries according to the total COVID-19 cases reported till 22 April 2020 [4,5] as depicted in Figure 3, and Figure 4 respectively.

## 3. Methodology

We performed following three analytical studies in this paper–a. correlation analysis to identify how human coronavirus spreading and its fatality are related to factors such as, external temperature, sunshine, rainfall, population, area, and density. b. finding importance of social isolation factor (*“f”*) to restrict the spread of COVID-19, and c. development of univariate LSTM models to forecast total death and total cases globally or country-wise (choice-based) and their performance comparison. 

The overall process (methodology) includes [56,57,58]–a. data collection/data simulation, b. data pre-processing, c. statistical analysis and data visualization, d. algorithm selection for LSTM model development, e. model training and testing, f. model evaluation, and g. model reusability.

### 3.1. Data Collection

In this study, we used two types of datasets—a. real datasets available in “ourworldindata.org” for timeseries forecasting and data visualization, and b. simulated dataset. For univariate timeseries forecasting, we used “ourworldindata.org” datasets (total cases and total death) from 1 January 2020 to 22 April 2020 for the whole world and afflicted specific countries separately for individual processing.

In contrast, we used two categories of simulated data–one for correlation analysis (“simulated_data_1”) and another (“simulated_data_2”) for the proposed algorithm in Section 3.8. The former simulated dataset (“simulated_data_1”) consisted of 18 features, such as “Temp-Jan”, “Temp-Feb”, “Temp-Mar”, “Rainfall-Jan”, “Rainfall-Feb”, “Rainfall-Mar”, “Sunshine-Jan”, “Sunshine-Feb”, “Sunshine-Mar”, “Population”, “Area”, “Population Density”, “Case-Jan”, “Case-Feb”, “Case-Mar”, “Death-Jan”, “Death-Feb”, “Death-Mar”, and “Country”. The measurable sensor data related to average external temperature (°C), average rainfall (ins) and daily sunshine (hrs.) were collected from “weather2visit.com” [59]. The approximated data related to the area, population, and population density (km^2^) were collected from “wikipedia” [60]. The simulated data was formed integrating facts from the following countries–China, Italy, Spain, Germany, Iran, Switzerland, South Korea, Belgium, Netherlands, Austria, Singapore, Malaysia, France, Australia, United States, United Kingdom, and Portugal as during January, 2020-March, 2020 mentioned countries were among the topmost risk zone. The features of the “simulated_data_1” has been described in Table 2.

We did a statistical analysis of other simulated data (“*simulated_data_2*”) with the following five features—a. count of days to run the simulation (“days”), b. assumed population (“population”), c. spreading factor (“spread_factor”), d. initial afflicted people (“initial_afflicted”), and e. total number of days to recover (“days_to_recover”) from COVID-19 to visualize the importance of social distancing by flattening the curve of afflicted population over days as described in Section 4. 

The datasets are as described in Table 3. All the simulated datasets are available in the repository as mentioned in the “Supplementary Materials” along with python codebase to reproduce the results.

### 3.2. Data Processing

Collected data are categorized among two groups–continuous and categorical. Accumulated data in this research are labeled. Downloaded data from “ourworldindata.org” are inconsistent with missing values. We utilized data mining techniques to filter data samples from the dataset, discard samples containing outliers, pattern discovery, calculation of feature correlation, feature selection, and noise removal. Data processing combines three steps as stated below [56,57,58]:

- Data preprocessing includes-data integration, removal of noisy data that are incomplete and inconsistent, data normalization and feature scaling, encoding of categorical data, feature selection after correlation analysis, and split data for training and testing an LSTM model.
- Training of a LSTM model and test its accuracy with loss functions as described in Section 3.5.
- Data postprocessing includes-pattern evaluation, pattern selection, pattern interpretation, and pattern visualization.

In this experiment, we have used “Python 3.x” language libraries for data processing, as described in Table 4. We established a python environment using anaconda distribution and “Spyder IDE” for developing python-based deep learning application. We used traditional “Keras” library with “TensorFlow” backend for LSTM model development, training, and testing.

### 3.3. Statistical Analysis 

In this study, we performed following two statistical approaches–hypothesis testing and correlation analysis. Hypothesis testing is a statistical method that is used in achieving statistical decisions using trial data. The critical parameter of hypothesis testing is the null hypothesis (**H_0_**), that tells there is nothing different or unique about the data. On the contrary, the alternative hypothesis (**Ha**) directly contradicts **H_0_**. The confidence factor or value of significance (α) is used to decide whether to accept or reject an **H_0_**. The value of α is usually kept as 0.05 or 5%, as 100% accuracy is impossible to achieve whether to accept or reject an **H_0_**. Popular, widely used hypothesis testing method, and a short description is demonstrated in Table 5. For the testing method, resultant probability value (*P-value*) is compared with “α” to accept or reject a null-hypothesis [56,57,58]. 

Example:

**Hypothesis** **(H_0_).** *Time series has a unit root (non-stationary). It has some time dependent structure.*

**Hypothesis** **(H_a_).** *The null hypothesis is rejected. It suggests that the time series does not have a unit root (stationary). It does not have time-dependent structure, and α = 5% or 0.05.*

Covariance (COV(x,y)) is a property of a function to retain its form when its variables are linearly transformed. It helps to measure correlation (r_xy_) that measures the strength of the linear relationship between two variables.

corr(x,y)=COV(x,y)/(σx∗σy), where−1<r<+1

The “sign” shows the direction of the relationship among two variables x and y. Table 6 shows the meaning of different |r| values. If two variables are strongly correlated, it is recommended to select any one of them during feature selection. Pearson’s correlation coefficient is used to summarize the strength of the linear relationship between two variables in normal distribution and spearman’s correlation is used to calculate the non-linear relationship between two variables. The used statistical methods are described in Table 7 [56,57,58,61]. 

### 3.4. LSTM Modelling

**Figure 5:**
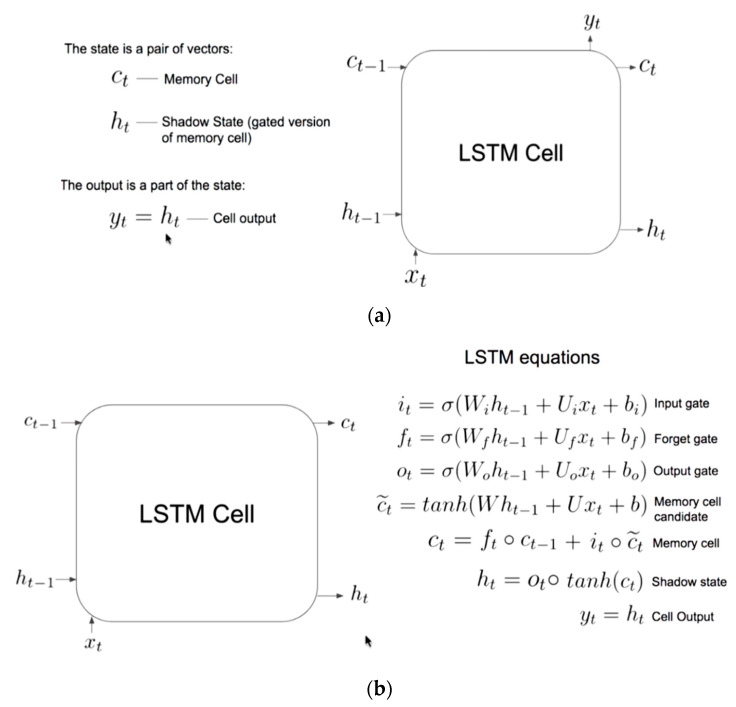
(**a**) A vanilla LSTM cell; (**b**) Equations of a vanilla LSTM cell.

The long short-term memory networks (LSTM) [63] are applied in long term dependencies, such as timeseries forecasting, handwriting recognition, speech detection, and anomaly detection in network traffic. LSTMs are a special kind of RNN and used in the field of deep learning. An LSTM model has a chain-like structure (a cell, an input gate, an output gate and a forget gate), but the repeating module has a different structure. Unlike standard feedforward neural networks, LSTM has feedback connections. LSTM networks are well-suited to classify, process, and make predictions based on timeseries data. They are used to overcome following two problems associated with the RNN–exploding gradients, and vanishing gradients. There are different types of LSTM models (univariate, multivariate, multi-step, and multivariate multi-step) which can be used for each specific type of timeseries forecasting problem. In this study, we have used univariate LSTM models, such as vanilla, stacked, bidirectional, and multilayer, for timeseries forecasting. The sates of a vanilla LSTM model are summarized below and illustrated in Figure 5.

**Step#1:** What we need to forget? Identify that information which are not required and must be thrown away from the cell state. This decision is made by a sigmoid layer called as forget gate layer (*“f_t_”*).

**Step#2:** What new information we are going to add to our cell state? A sigmoid gate called the “input gate layer” decides which values will be updated (*“o_t_”*). Next, a “tanh” layer creates a vector of new candidate values, that could be added to the state.

**Step#3:** Combine step#1 and step#2 to achieve a new cell state (*“c_t_”*), and

**Step#4:** Finally, receive the output (*“h_t_”*).

In this study, we have selected below six LSTM models for timeseries analysis and forecasting:

***a***. ***Vanilla LSTM Modelling in Keras:***
  model = Sequential ()
  model.add (LSTM (50, activation =‘relu’, input_shape = (3, 1)))
  model.add (Dense (1))
***b***. ***Stacked LSTM Modelling in Keras:***
  model = Sequential ()
  model.add (LSTM (100, activation =‘relu’, return_sequences =True, input_shape = (3, 1)))
  model.add (LSTM (100, activation =‘relu’))
  model.add (Dense (1))
***c***. ***Bidirectional LSTM Modelling in Keras:***
  model = Sequential ()
  model.add (Bidirectional (LSTM (100, activation =‘relu’), input_shape = (3, 1)))
  model.add (Dense (1))
***d***. ***Multilayer LSTM 1 Modelling in Keras:***
  model = Sequential ()
  model.add (LSTM (units = 92, return_sequences = True, input_shape = (3, 1)))
  model.add (Dropout (0.2))
  model.add (LSTM (units = 92, return_sequences = True))
  model.add (Dropout (0.2))
  model.add (LSTM (units = 92, return_sequences = True))
  model.add (Dropout (0.2))
  model.add (LSTM (units = 92, return_sequences = False))
  model.add (Dropout (0.2))
  model.add (Dense (units = 1))
***e***. ***Multilayer LSTM 2 Modelling in Keras:***
  model = Sequential()
  model.add(LSTM(units = 100, return_sequences = True, input_shape = (3, 1)))
  model.add(Dropout(0.2))
  model.add(LSTM(units = 100, return_sequences = True))
  model.add(Dropout(0.2))
  model.add(LSTM(units = 100, return_sequences = True))
  model.add(Dropout(0.2))
  model.add(LSTM(units = 100, return_sequences = True))
  model.add(Dropout(0.2))
  model.add(Dense(units = 1))
***f***. ***Multilayer LSTM 3 Modelling in Keras:***
  model = Sequential()
  model.add(LSTM(units = 50, return_sequences = True, input_shape = (3, 1)))
  model.add(Dropout(0.2))
  model.add(LSTM(units = 50, return_sequences = True))
  model.add(Dropout(0.2))
  model.add(LSTM(units = 50, return_sequences = True))
  model.add(Dropout(0.2))
  model.add(LSTM(units = 50, return_sequences = False))
  model.add(Dropout(0.2))
  model.add(Dense(units = 1))

**Note:**

- The “Dropout layer” refers to dropping out units (both hidden and visible neuron) in a neural network.
- There are three deep learning model optimizers for hyperparameter tuning and cross validation– a. Adaptive gradient (ADAGARD), b. RMSProp (adds exponential decay), and c. ADAM. In this study, we used “ADAM” optimizer.
- Mean square error (MSE), mean absolute error (MAE), “categorical_crossentropy”, “binary_crossentropy”, residual forecast error/forecast error, forecast bias/mean forecast error, root mean square error (RMSE), and “R^2^-score” are different determining methods for model loss, but we used “MSE”, “MAE”, “RMSE”, forecast bias, and “R^2^-score”.
- “Dense layer” is the regular deeply connected neural network layer.
- “ReLU” stands for rectified linear unit. It is a type of activation function. Mathematically, it can be defined as y = max (0, x), where x > 0. Its convergence is faster. It is fast to compute. It is sparsely activated.
- LSTM units can be trained in a supervised fashion, on a set of training sequences, using an optimization algorithm, such as gradient descent, combined with backpropagation through time to compute the gradients needed during the optimization process, to change every weight of the LSTM network in proportion to the derivative of the error (at the output layer of the LSTM network) with respect to corresponding weight.

### 3.5. Model Training and Testing

The steps applied to train and test an LSTM model in this study, are described as below:

- Importing of python libraries
- Load data from repository
- Data pre-processing:
  ○ remove missing value from the loaded data
  ○ encode categorical features
  ○ check distribution of data and features
  ○ correlation analysis among features and feature scaling if required
- Feature scaling with “MinMaxScaler (feature_range = (0, 1))”
- Split the univariate sequence into samples
- Split data for training (97%) and testing (3%)
- Create LSTM models as described in Section 3.4.
- Compile the model with optimizer = “adam”, loss =“mse”, metrics = [“acc”]
- Train the model with epochs = 100, batch_size = 10, validation_split = 0.05
- Use “ADAM” optimizer for model tuning
- Evaluate model performance with accuracy and loss function after inverse transformation of the predicted feature.
- Execute the model for five times and then calculate the average of performance metrics as described in Section 3.5, and predicted value. It helps prove the testing rate and increase the validity of timeseries analysis.

**Note:**

- Univariate sequences are timeseries data of total cases, and total death for the world or individual countries. In this study, we have considered univariate timeseries data of the world for both training and testing of LSTM models, but the same model can be extended to use for individual countries.
- The “acc” refers to accuracy in **metrics** = [“acc”] of the corresponding LSTM model.

### 3.6. Model Performance Evaluation

Developed univariate LSTM models for timeseries forecasting are evaluated with below metrices:

- Regression metrices: mean absolute error (MAE), mean squared error (MSE), root mean square error (RMSE), forecast bias, and R^2^ regression metric.

MAE is the easiest error metric used in the regression problem following the formula:

(1) $$MAE=\frac{1}{n}\sum^{\;}|Y-\hat{Y}|,\;where\;Y=actual\;value\;and\;\hat{Y}=predicted\;value$$

MSE squares the difference of actual and predicted output before adding them all instead of using the absolute value following the formula:

(2) $$MSE=\frac{1}{n}\sum^{\;}{\left(Y-\hat{Y}\right)}^{2},\;where\;Y=actual\;value\;and\;\hat{Y}=predicted\;value$$

RMSE is the square root of the calculated mean squared error (MSE).

Forecast bias can be wither positive or negative. The forecast bias is calculated directly as the mean of the forecast error. A mean forecast error value other than zero suggests a tendency of the model to over forecast (negative error) or under forecast (positive error). As such, the mean forecast error is also called the forecast bias. If forecast error = 0, then no error, or perfect skill for that forecast. If forecast bias < 0, then over forecast and if forecast bias = 0 or close to zero, then the model is unbiased.

*forecast_error = (expected_value − predicted_value)* *forecast_bias = mean (forecast_error)* (3)

R^2^ regression metric has been used for the explanatory purpose to provides an indication of the fitness in predicted output values to the actual output values. It is calculated with a formula having numerator as MSE and the denominator as the variance in Y values. The R^2^ signifies how much variance of the data is explained by the model. The R^2^ = 0.90 means that 0.10 of the variances cannot justify by the model, when the logical case is R^2^ = 1, then the model completely fit and explained all variance. The calculation of R^2^ > 1 represents an abnormal case that has no logical meaning, and it may result from the small sample size.

(4) $$R^{2}=1-\frac{\frac{1}{n}\sum_{i=1}^{n}{\left(Y-\hat{Y}\right)}^{2}}{\left(\frac{1}{n}\sum_{i=1}^{n}(Y-{\bar{Y}}^{2}\right)},\;where=actual\;value,\;\hat{Y}=predicted\;value,\;and\;\bar{Y}=mean\;value$$

### 3.7. Model Store and Reuse

We saved our final trained LSTM model in a file and restored it to reuse, either by comparing the model with other models or by testing the model on a new or updated data. The process of storing the model is called serialization, and restoring the model is called deserialization. It can be done in two ways, as described in Table 8. The pickled model can be stored in the database for distributed access.

### 3.8. Algorithm Design to Find the Importance of Social Distancing

In this research, we studied the importance of social distancing by flattening the curve of afflicted population over specific days, with a spreading factor (*“f”*) of 0 < *f* ≤ 5 [6]. The spread factor is used to determine the transmission rate of a virus [6]. 

If *“f”* = 0 then no spreading, else one infected person can infect up to 3–5 people daily, in maximum [6]. The recovery from COVID-19 takes a maximum of 7–10 days [6]. Therefore, we have selected the value of “days_to_recover” as 10. In the proposed algorithm, we assumed that no patient has died. The “days” feature can be contemplated as a “lockdown” period. The Algorithm 1 we used for analysis is described below –
**Algorithm 1.** Importance of social distancing by flattening the curve of afflicted population over specific days  ***Step 1:** Initialize necessary parameters as follows to create a simulated town infected with COVID-19 –*      days = 100/*lockdown days*/      population = 200,000/*population of the town*/      spread_factor = 0.25/*COVID-19 transmission rate (0 < f ≤ 5) */      days_to_recover = 10/*maximum recovery days from COVID-19*/      inital_afflicted_people = 5/*initial infected people of the town with COVID-19*/  ***Step 2:** Initialize a data frame (“town”) for the simulated town with the following four features –*      id = range(population)/* id € (0- population) */      *infected = false*
      *recovery_day = none*      *recovered = false*
  ***Step 3:** Initialize the initial cases (“initialCases”) with **inital_afflicted_people** variable,*     *update corresponding **infected** feature as **true**, and*
     *update **recovery_day** feature with **days_to_recover** variable*  ***Step 4:** Initialize the initial active cases (“active_cases”) with **initally_afflicted** variable and*     *initial recovered cases (“recovery”) with **0**.*  ***Step 5:***      *for day = 1 to days do*          *Step 5.1: Mark the people of **town** data frame, who have recovered on current **day***
              *- update the feature **recovery_day** as **True** and **infected** feature as **False***
              *if they have crossed **days_to_recover** else **ignore**.*
          *Step 5.2: Calculate the number of people who are afflicted today with **spread_factor***              *- calculate number of people infected in the **town** data frame based on*
              *feature **infected** = True*              *-multiply the count of total infected people with **spread_factor** to*
              *calculate total possible cases of infected people on current **day***          *Step 5.3: Forget people who were already infected in cases of current **day***          *Step 5.4: Mark the new cases as afflicted, and their recovery day by updating*
              ***active_cases** and **recovery** lists of the **town** data frame.*  ***Step 6:** Repeat the step 5 for **spread_factor** = 0.25 to 5.0 and plot every distribution graph of **active_cases** over **days**.*

***Note:***

- We plotted distribution graph of active cases over days for the following set of “f” values: [0.25, 0.5, 0.75, 1.0, 2.0, 3.0, 4.0, 5.0] with the initialized parameters at Step 1.
- The algorithm was implemented with “simulated_data_2”.
- The worst-case time complexity of the algorithm is O(N^2^), where N = problem size.

## 4. Results and Discussion

**Figure 6:**
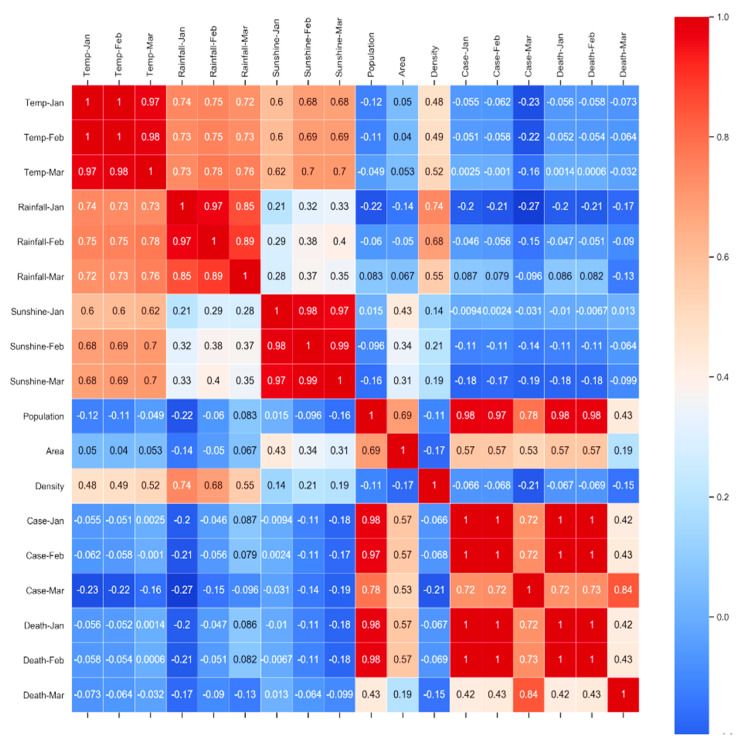
Correlation heatmap of simulated data (“simulated_data_1”) to check feature correlation.

The correlation analysis of the simulated data (“*simulated_data_1*”) is depicted in Figure 6. The resultant correlation heatmap of simulated data is a well-accepted data visualization method among machine learning communities, and it illustrates the magnitude of a phenomenon as color in two dimensions. Here, the variation in color is the value of correlation factor “r” which is giving understandable visual cues about how the phenomenon is clustered or varies over space. The code of the color is changing according to the values of “r”, from a weak correlation to the strong correlation. The color bar beside the correlation matrix is signifying that color change following “r” values, where −1 < r < +1 as described in Section 3.3. 

We excluded the feature “country” from correlation study. The correlation study was conducted to investigate how infected cases and death are related to external temperature, sunshine, and precipitation! Correlation factor |r| > 0.6 represents a strong correlation according to Table 5. 

In this study, we represented the relation between total population (p), cases (c), and death (d) with the following functions *(“f”)* –

*c = f(p)*, and *d = f(c), where p > 0, c > 0, d > 0, and p, c, d are natural numbers (N). Hence, d = f(f(p)).*

Generally, *dc/dt ≥ 0, dd/dt ≥ 0, and dp/dt > 0, where “t” is the time and t > 0*.

*Let, p’* is the total infected population, where *p’€ p.*

*Let, c = f(p’)* is a function defined on an interval *[a, a + h]*, where “a” is the initial infected population, “h” is the newly infected population, *{a, h} € p’, a ≥ 0, and h < p’*. 

Therefore, the instantaneous rate of change of “*c*” at “a” is its derivative –

$$f^{\prime }\left(a\right)=\underset{h\to 0}{\lim}\frac{f\left(a+h\right)-f\left(a\right)}{h}$$

Hence, for small change in “h”, *f’(a)* approximates to *(f (a + h) − f (a))/h*. Subsequently, it can be derived that –

*dc/dt = dp′/dt ≥ 0*

**Figure 7:**
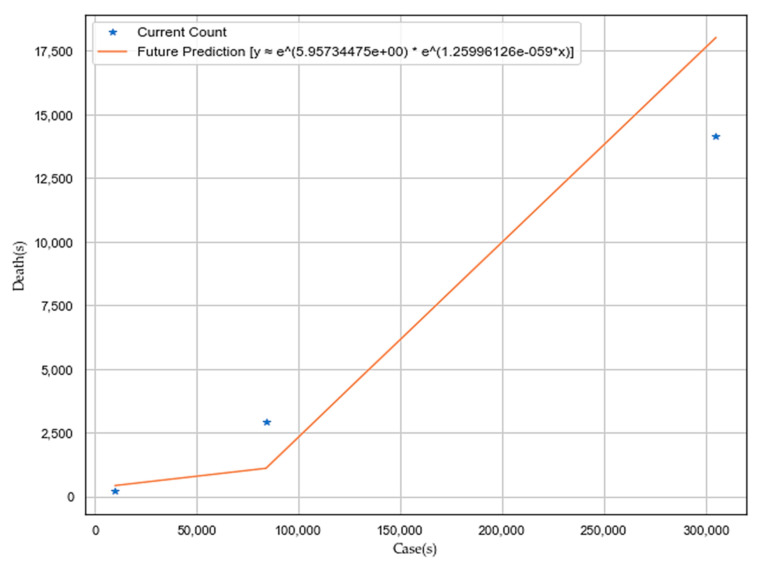
Exponential regression plot to show death increases with number of cases.

The correlation analysis, as depicted in Figure 6, is exhibiting that COVID-19 does not have any dependency on external temperature, sunshine, and precipitation. It is genuinely a community disease. Death is highly correlated (|r| > 0.8) to the number of cases rather than the weather (external temperature, sunshine, and precipitation), as depicted in Figure 7. We performed exponential regression analysis to plot increase in death (*Y*-axis) with an increase in the number of cases (*X*-axis) as depicted in Figure 7, and the obtained equation of an approximated exponential curve is: *Y = e*∧(5.95734475e+00) * e∧(1.25996126e-05*X)**.**

The total cases are highly related (|r| > 0.7) to the population, as depicted in Figure 6. If the number of populations increases, the number of new deaths also increases due to the high correlation value of |r|, as depicted in Figure 6. Therefore, social distancing or social isolation is one of the primary keys to stop its spreading. Countries with high population density, such as Bangladesh, Singapore, Pakistan, and India, have a high chance of getting afflicted by COVID-19 very drastically until controlled from the beginning. Hence, social isolation, lockdown, social distancing are significant in this regard to stop the spreading of COVID-19 at the community level. 

That is why, many countries have been locked down, and people are being asked to stay at home. It might have a chance to slow down the spread of the COVID-19 by flattening the curve of afflicted population over days and relaxing pressure on the healthcare system. It is one of the essential measures to restrict the fatality rate of COVID-19. Besides the decision of lockdown, ordinary people should understand its importance as the human coronavirus is highly contagious.

**Figure 8:**
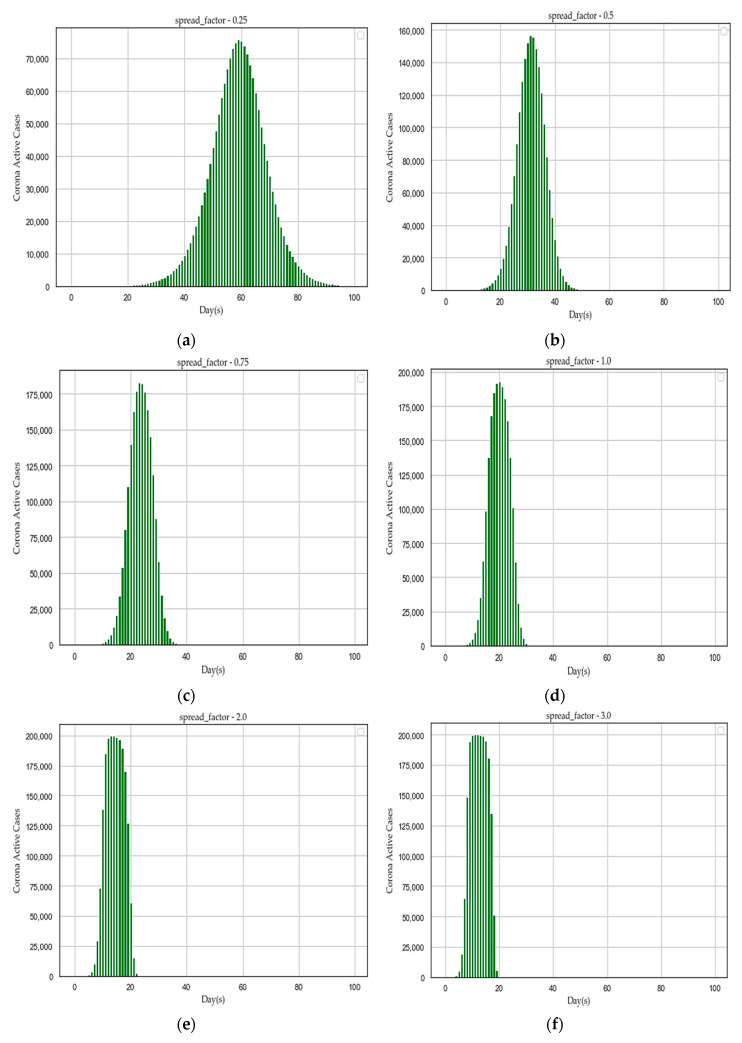

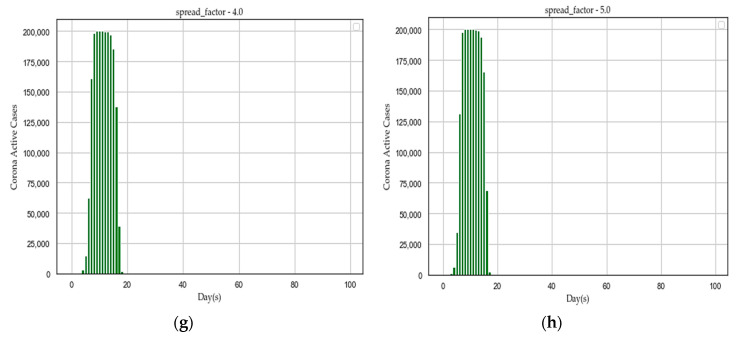
Flattening the distribution graphs of active cases over days by reducing human coronavirus spreading with different *“f”* values, such as (**a**) f = 0.25; (**b**) f = 0.50; (**c**) f = 0.75; (**d**) f = 1.00; (**e**) f = 2.00; (**f**) f = 3.00; (**g**) f = 4.00; and (**h**) f = 5.00.

We hypothesized that social isolation or social distancing might restrict the spreading of the human coronavirus as it may slow down the spread factor (*“f”*). To prove the assumed hypothesis, we proposed an algorithm in Section 3.8. After executing the algorithm with simulated data (“*simulated_data_2*”), we plotted different distribution graphs of “active cases” (*Y*-axis) over the number of lockdown “days” (*X*-axis), for the following set of spread factor (*“f”*) values: *[0.25, 0.5, 0.75, 1.0, 2.0, 3.0, 4.0, 5.0]* as depicted in Figure 8. The spread factor (*“f”*) with the lowest value of 0.25 has produced a nice gaussian distribution in Figure 8. With increasing spread factor (*“f”*), active cases are growing high as compared to lockdown period, as described in Table 9. If the average load of active cases goes high in short span of days as described in Table 9, the healthcare sector may collapse to cope up and unable to provide adequate treatment to infected patients. Therefore, the recovery rate may become very low and death rate may increase. The figures (Figure 8) are illustrating that social isolation or social distancing has a significant impact on flattening the curve of afflicted population over days to alleviate sudden pressure on the existing capacity of the healthcare system. 

**Figure 9:**
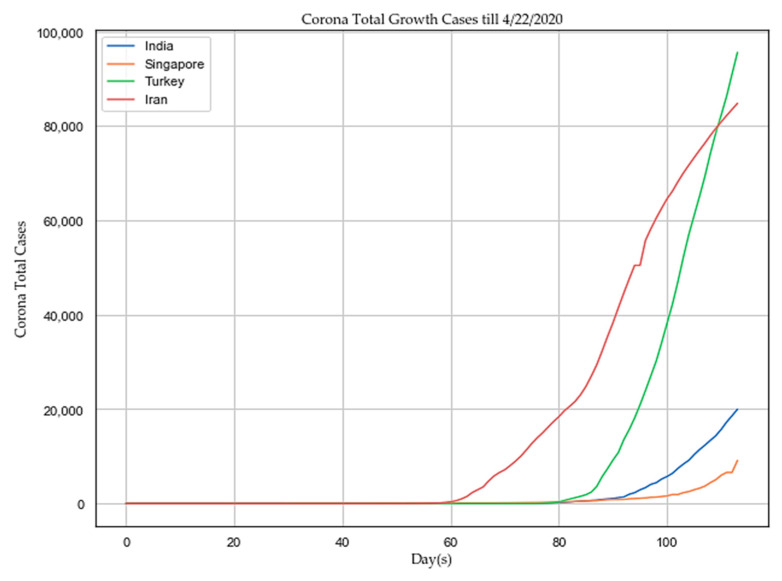
Trend analysis of total reported cases in four Asian countries.

India implemented its first lockdown on 23 March 2020 to 13 April 2020 (21 days) and the second lockdown until 3 May 2020. The trend of total reported cases has been compared between four Asian countries, such as India, Singapore, Iran, and Turkey, till 22 April 2020 as depicted in Figure 9. The trend is showing that successful lockdown might have a chance to slow down the spreading of the human coronavirus in India and Singapore. As per the study at “John Hopkins University”, the human coronavirus growth rate in India is declining consistently by flattening the curve of case doubling due to the first phase of lockdown [14]. 

We downloaded four types of timeseries data from “ourworldindata.org” as follows–a. the total number of cases, b. total deaths, c. new confirmed cases, and d. new deaths. We performed hypothesis testing on the timeseries data to check whether they are stationary or not, following Table 5. The result is described in Table 10. 

We analyzed the performance of six LSTM models as described in Section 3.4 on the following two datasets—a. the total number of cases, and b. total deaths, available in “ourworldindata.org” to forecast probable total infected cases and death in advance. The designed models can be used to forecast total infected cases and total deaths of any selected countries individually, available in “ourworldindata.org”. We processed data from 1 January 2020, to 22 April 2020 as described in Section 3.5. Total 97% of the data utilized to train the models and the remaining 3% data used for testing (total 110 future predictions) the performance of the models. 

**Figure 10:**
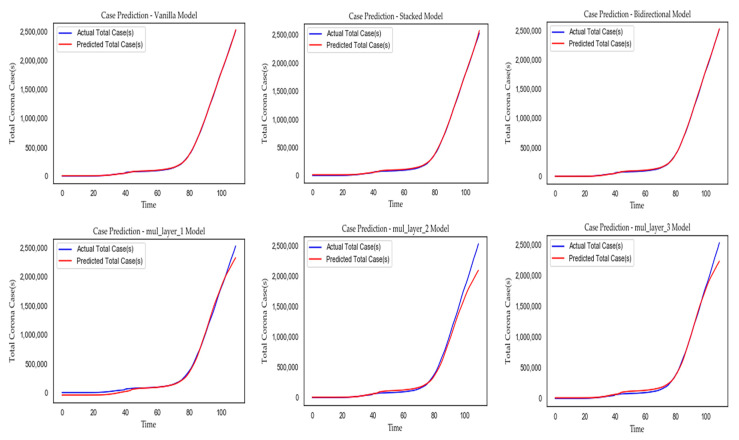
Comparing the calibration of the LSTM models to forecast total cases of the “World”.

**Figure 11:**
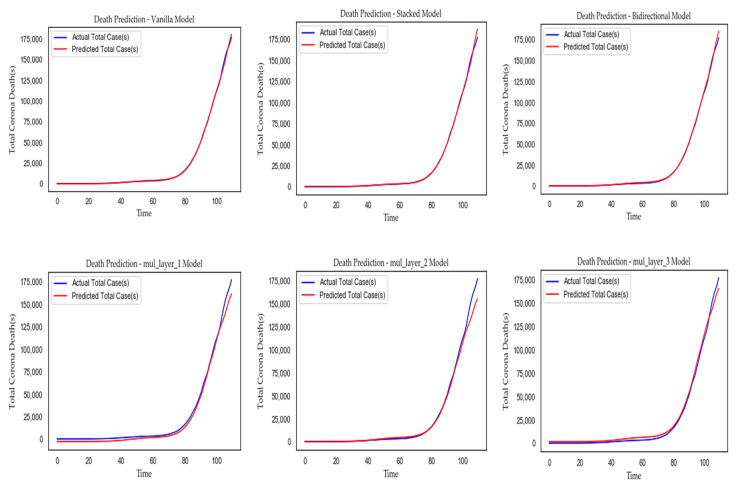
Comparing the calibration of the LSTM models to forecast total deaths of the “World”.

We executed training and testing of individual models for 5 times, then took the average of corresponding performance metrics, and predicted values. The average performance results of different LSTM models are described in Table 11 and Table 12 respectively, and corresponding model calibrations are depicted in Figure 10 and Figure 11, respectively. According to the result, no single model is 100% accurate, and they tend to either over-forecast or lower forecast. The vanilla, stacked, and bidirectional LSTM models performed better than multilayer LSTM models. In this study, we focused only on the general trend of data, and that might be the reason to over-forecast. The forecasting may help us to be aware of upcoming unwanted situations and take necessary actions in advance to mitigate it.

For verification, we trained our vanilla, stacked, and bidirectional LSTM models with Indian dataset available in “ourworldindata.org” from 1 January 2020 to 23 March 2020. The focus was to forecast an approximate total number of cases after 21 days starting 23 March 2020, as the first lock-down period of India ended on 13 April 2020. We executed individual models for 5 times, then took the average of total predicted cases. Once forecasting was completed, we verified whether lock-down (social distancing/social isolation) had any impact on lowering the spread of the human coronavirus. The result showed that without lock-down, India could cross 0.2 million of total corona cases on 14 April 2020. Therefore, it supports our assumed hypothesis that social isolation/social distancing is one of the main criteria to fight against COVID-19.

## 5. Conclusions

The statistical correlation study proved that COVID-19 does not depend on external weather factors, such as external temperature, sunshine, and precipitation. It depends on the population and its density mostly. Therefore, it is considered as a community disease. This research verified our assumed hypothesis that social isolation/social distancing might restrict the spreading of the human coronavirus by diminishing its spread factor. The forecasting of probable new corona cases and death count with proposed LSTM models in this study may help to take necessary actions in advance to control the upcoming undesirable health crisis. SARS-CoV-2 can infect people of all ages, but people who have pre-existing medical conditions such as COPD, CVDs, diabetes, hypertension, cerebrovascular disease, and cancer are more susceptible to become severely sick with the viral infection. Complete data related to different health factors, age, sex, health history of COVID-19 infected patients are still not available in public to conduct more detailed research. 

In the future, the accuracy of the LSTM forecasting can be improved after considering additional needed parameters rather than relying on univariate trend of timeseries data. eHealth with information and communication technologies (ICT) [65], may open a new direction in COVID-19 research and remote patient monitoring after collecting necessary health and wellness data through standard sensors, questionnaires and followed by, train a decision support system (DSS) for tailored recommendation generation.

## Figures and Tables

**Table 1:** Propagation of human coronavirus through air [19,47,48,49,50,51,52].

No	Size	Transmission Distance
1	Larger respiratory droplets (>5–10 μm diameter)	Travel only short distances, generally < 1 m, but in extraordinary cases up to 4 m
2	Virus-laden small (<5 μm diameter) aerosolized droplets (droplet nuclei)	Travel long distances, >1 m
3	Combinations of an individual patient’s physiology and environmental conditions, such as humidity and temperature, the gas cloud, and its payload of pathogen-bearing droplets of all sizes	Travel 7–8 m
4	Strong airflow from the air conditioner	Distance above 1 m

**Table 2:** Feature description of “simulated_data_1”.

No.	Features	Description
1	Temp-Jan	Average temperature of the country in January 2020 [59]
2	Temp-Feb	Average temperature of the country in February 2020 [59]
3	Temp-Mar	Average temperature of the country in March 2020 [59]
4	Rainfall-Jan	Average rainfall of the country in January 2020 [59]
5	Rainfall-Feb	Average rainfall of the country in February 2020 [59]
6	Rainfall-Mar	Average rainfall of the country in March 2020 [59]
7	Sunshine-Jan	Average sunshine of the country in January 2020 [59]
8	Sunshine-Feb	Average sunshine of the country in February 2020 [59]
9	Sunshine-Mar	Average sunshine of the country in March 2020 [59]
10	Population	Total population of the country [60]
11	Area	Total area of the country [60]
12	Population Density	Population density of the country [60]
13	Case-Jan	Total infected cases of the country in January 2020 [5]
14	Case-Feb	Total infected cases of the country in February 2020 [5]
15	Case-Mar	Total infected cases of the country in March 2020 [5]
16	Death-Jan	Total deceased of the country in January 2020 [5]
17	Death-Feb	Total deceased of the country in February 2020 [5]
18	Death-Mar	Total deceased of the country in March 2020 [5]
19	Country	Name of the country selected for analysis

**Table 3:** Description of selected datasets.

No	Name	External Source	Purpose	Description
1	COVID-19 datasets	www.ourworldindata.org [5]	Univariate LSTM forecasting	It is containing world-wide and country specific data, such as total cases, death, recoveries.
2	Simulated_data_1	www.weather2visit.com [59], www.wikipedia.com [60]	For correlation analysis	It is containing features, such as external temperature, rainfall, sunshine, population, infected cases, death, country, population, area, and population density of the past three months-January, February, and March
3	Simulated_data_2	Not available	For analyzing our proposed algorithm	Key variables used in the algorithm are as follows: days = 100, population = 200,000, days_to_recover = 10, inital_afflicted_people = 5, and spread_factor = [0.25, 0.5, 0.75, 1.0, 2.0, 3.0, 4.0, 5.0]

**Table 4:** Python libraries for data processing [61].

No.	Libraries	Purpose
1	Pandas	Data importing, structuring and analysis
2	NumPy	Computing with multidimensional array object
3	Matplotlib	Python 2-D plotting
4	SciPy	Statistical analysis
5	Seaborn, plotly	Plotting of high-level statistical graphs
7	Keras with TensorFlow	LSTM model development, training, and testing

**Table 5:** Hypothesis testing method [62].

Method	Description
Augmented Dickey-Fuller test	To test if a timeseries is stationary or non-stationary

**Table 6:** Significance of regression coefficient (r).

|r| Value	Meaning
0.00–0.2	Very weak
0.2–0.4	Weak to moderate
0.4–0.6	Medium to substantial
0.6–0.8	Very strong
0.8–1	Extremely strong

**Table 7:** Statistical analysis methods on the selected datasets.

No.	Methods	Purpose
1	Mean, standard deviation	Distribution test
2	Covariance, correlation	Association test
3	Histogram, line, bar, Scatter	Distribution plot
4	Quantile analysis	Outlier detection

**Table 8:** LSTM model store [61,64].

Method	Implementation
Pickle string	Import pickle library
Pickled model	Import joblib from sklearn.externals library

**Table 9:** Effect of spreading factor *(“f”)* to flatten the curve of active cases.

*“f”*	Peak Active Cases	Span of Active Cases (Days)	Treatment Duration (Days)	Maximum Load (Week)	Avg Load (Patient/day)
0.25	70,000–80,000	1–100	100	7–10	Moderate
0.50	140,000–160,000	1–50	50	4–5	Medium
0.75	175,000–190,000	1–40	40	3–4	High
1.00	175,000–200,000	1–36	36	2–4	High
2.00	200,000	1–23	23	2–3	Very High
3.00	200,000	1–19	19	2–3	Very High
4.00	200,000	1–17	17	1–2	Very High
5.00	200,000	1–18	18	1–2	Very High

**Table 10:** Result of hypothesis testing of timeseries data.

Timeseries Data	Test Result	Nature of Data
Total_deaths	ADF Statistic: −4.763,824 *p*-value: 0.000,064 Critical Values: 1%: −3.498 5%: −2.891 10%: −2.582	Rejecting null hypothesis; no unit root and timeseries is stationary
New_deaths	ADF Statistic: −2.814,703 *p*-value: 0.056,204 Critical Values: 1%: −3.498 5%: −2.891 10%: −2.582	Fail to reject null hypothesis; the data has a unit root and data is non-stationary
Total_cases	ADF Statistic: 5.989,246 *p*-value: 1.000,000 Critical Values: 1%: −3.496 5%: −2.890 10%: −2.582	Fail to reject null hypothesis; the data has a unit root and data is non-stationary
New_cases	ADF Statistic: 2.771,519 *p*-value: 1.000000 Critical Values: 1%: −3.498 5%: −2.891 10%: −2.582	Fail to reject null hypothesis; the data has a unit root and data is non-stationary

**Table 11:** Average performance analysis of LSTM models to forecast total cases of the “World”.

LSTM Model(s)	MAE	MSE	RMSE	Forecast Error	|R^2^|	Compilation Time (ms)
Vanilla	8,968.244	98,168,777.193	9,908.016	121.883	1.0	110.0
Stacked	6,597.784	82,779,520.484	9,098.325	1,120.341	1.0	192.0
Bidirectional	7,130.149	74,807,857.322	8,649.154	1,454.284	1.0	194.0
Multi-Layer 1	37,438.048	2,338,577,178.93	48,358.838	−37,075.648	0.995	520.0
Multi-Layer 2	45,038.733	4,110,861,091.40	64,115.997	15,340.520	0.992	762.0
Multi-Layer 3	51,890.187	10,545,625,824.0	102,691.898	−45,213.395	0.970	680.0

**Table 12:** Average performance analysis of LSTM models to forecast total death of the “World”.

LSTM Model(s)	MAE	MSE	RMSE	Forecast Error	|R^2^|	Compilation Time (ms)
Vanilla	735.039	2,300,815.114	1,516.844	−120.177	0.99	104.0
Stacked	738.703	4,637,553.996	2,153.498	341.605	0.98	190.0
Bidirectional	660.818	1,114,423.658	1,055.663	394.884	0.99	191.0
Multi-Layer 1	3,573.872	30,177,345.174	5,493.391	−3,573.872	0.983	400.0
Multi-Layer 2	1,290.960	4,069,047.834	2,017.188	−708.400	0.998	407.0
Multi-Layer 3	3,108.016	52,959,914.784	7,277.356	−3,033.915	0.966	400.0

## Supplementary Materials

The following are available online at https://www.mdpi.com/1424-8220/20/11/3089/s1.
