# Supplementary material for: Statistical Explorations and Univariate Timeseries Analysis on COVID-19 Datasets to Understand the Trend of Disease Spreading and Death
Source: Sensors (Basel). 2020 May 29;20(11):3089. doi: 10.3390/s20113089 (PMC7308840; doi:10.3390/s20113089)
Supplement: Supplementary file 1 [file sensors-20-03089-s001.pdf]

sensors

MDPI

Supplementary Materials

Statistical Explorations and Univariate Timeseries Analysis on COVID-19 Datasets to Understand the Trend of Disease Spreading and Death

Ayan Chatterjee [1,*], Martin W. Gerdes [1] and Santiago G. Martinez [2]

[1] Department of Information and Communication Technology, Centre for e-Health, University of Agder, 4604 Kristiansand, Norway; martin.gerdes@uia.no

[2] Department of Health and Nursing Science, Centre for e-Health, University of Agder, 4604 Kristiansand, Norway; santiago.martinez@uia.no

* Correspondence: ayan.chatterjee@uia.no

Supplementary Materials: To reproduce the result, the codebase can be downloaded from GitHub repository: https://github.com/ayan1c2/Timeseries_LSTM_COVID19.git.
